# Retinal structure and function preservation by polysaccharides of wolfberry in a mouse model of retinal degeneration

**DOI:** 10.1038/srep07601

**Published:** 2014-12-23

**Authors:** Ke Wang, Jia Xiao, Bo Peng, Feiyue Xing, Kwok-Fai So, George L. Tipoe, Bin Lin

**Affiliations:** 1 Department of Anatomy, Li Ka Shing Faculty of Medicine, The University of Hong Kong, Pokfulam, Hong Kong; 2 Department of Ophthalmology, Li Ka Shing Faculty of Medicine, The University of Hong Kong, Pokfulam, Hong Kong; 3 Department of Immunobiology, Institute of Tissue Transplantation and Immunology, Jinan University, Guangzhou, China; 4 GHM Institute of CNS Regeneration, Jinan University, Guangzhou, China

## Abstract

Retinitis pigmentosa (RP) is a heterogeneous group of inherited disorders caused by mutations in a variety of genes that are mostly expressed by rod cells, which results in initial death of rod photoreceptors followed by gradual death of cone photoreceptors. RP is currently untreatable and usually leads to partial or complete blindness. Here, we explored the potential neuroprotective effects of polysaccharides of wolfberry, which are long known to possess primary beneficial properties in the eyes, on photoreceptor apoptosis in the rd10 mouse model of RP. We found that these polysaccharides provided long-term morphological and functional preservation of photoreceptors and improved visual behaviors in rd10 mice. Moreover, we demonstrated that polysaccharides exerted neuroprotective effects through antioxidant, anti-inflammatory and anti-apoptotic mechanisms. Furthermore, we identified that polysaccharides modulated inflammation and apoptosis partly through inhibition of NF-κB and HIF-1α expressions, respectively. Overall, we demonstrated the synergistic protective effects of polysaccharides in preserving photoreceptors against degeneration in rd10 mice. Our study provides rationale and scientific support on using polysaccharides of wolfberry as one supplementary treatment of RP patients in the future.

Retinal photoreceptors are the primary sensory neurons that respond to light and send signals to other neurons by a change in their membrane potentials when they absorb photons. Vertebrate retinas contain two classes of photoreceptors, rods and cones. Rods are responsible for vision under dim illumination, whereas cones are responsible for conventional image-forming daylight and color vision. Millions of people worldwide lose their vision every year as a result of a retinal degeneration disorder known as retinitis pigmentosa (RP). RP is a heterogeneous group of inherited retinal degeneration characterized by an initial loss of night vision as a result of the dysfunction and death of rod photoreceptors in early adolescence, followed by a progressive degeneration of cone photoreceptors^1^. RP is currently untreatable and usually leads to partial or complete blindness. Although various approaches have been explored to preserve or replace photoreceptors in RP, including antioxidants, gene therapy, and stem cell therapy^2^,^3^,^4^,^5^,^6^, few effective treatments are currently available for RP patients.

Wolfberry (also known as *Lycium barbarum*) has been used as a traditional Chinese herbal medicine for thousands of years in the treatment and prevention of diseases such as liver dysfunction, and vision degeneration^7^,^8^,^9^. One of its most important bioactive components is *Lycium barbarum* polysaccharides-protein complex (LBP), which consists of six monosaccharides (galactose, glucose, rhamnose, arabinose, mannose, and xylose) and antioxidants^10^. Polysaccharides of wolfberry have been shown to possess a wide range of biological activities, including antioxidant, and anti-inflammatory effects^7^,^8^,^9^,^11^. In the eyes, we have previously shown that polysaccharides treatment preserves retinal ganglion cells in an experimental animal model of glaucoma and in ischemic retinas^11^,^12^. We have found that polysaccharides exert neuroprotection by down-regulating the receptor for advanced glycation end products (RAGE), endothelin-1 (ET-1), amyloid-β (Aβ) and advanced glycation end products (AGEs) in ischemic retinas, as well as by inhibiting oxidative stress and the c-jun N-terminal kinase (JNK) pathways, and increasing production of insulin-like growth factor-1 (IGF-1) in the retina after partial optic nerve transection. In the liver, we recently demonstrated that two bioactive components of wolfberry, l-arabinose and β-carotene, appear to exert the hepato-protective effects on hepatocytes by downregulating oxidative stress, inflammation, and apoptosis partially through a nuclear factor kappa B (NF-κB)-dependent pathway^13^. In the present study, we explored potential beneficial effects of polysaccharides of wolfberry on the pathological processes in RP retinas. For this purpose, we used rd10 mice, which is a well-characterized mouse model of RP caused by a mutation in the rod-specific gene that encodes the beta subunit of the rod phosphodiesterase-6 gene (Pde6b). Mutations in the Pde6b gene also cause RP in humans^14^. Therefore, rd10 mice provide an ideal model of progressive process in RP. In rd10 mice, initiation of rod photoreceptor degeneration begins around P18, with peak photoreceptor death occurring at P25^15^. We fed rd10 mice with polysaccharides starting from postnatal day 14 (P14) and killed animals at several time points to investigate the protective effect of polysaccharides on rod and cone apoptosis.

## Methods

### Animals and treatment

Wild-type (C57BL/6J), rd10 and *Cx3cr1^GFP/GFP^* mice were obtained from Jackson Laboratory (Bar Harbor, ME). Rd10 mice were backcrossed with *Cx3cr1^GFP/GFP^* mice, and the littermates from rd10/*Cx3cr1^+/GFP^* mice and rd10 mice were used for experiments. All experimental procedures were approved by the Committee on the Use of Live Animals in Teaching and Research at The University of Hong Kong and conducted in accordance with the ARVO statement for the use of animals. The Laboratory Animal Unit of the University of Hong Kong is fully accredited by the Association for Assessment and Accreditation of Laboratory Animal Care International (AAALAC international).

The preparation for polysaccharide extracts of wolfberry was the same as previously reported by us^11^,^16^. Oral administration was performed using a feeding needle with polysaccharides of 1 mg/kg or PBS once a day, starting from postnatal day 14 (P14) and continued through to P25, P29, or P41.

### Immunocytochemistry and confocal imaging

Animals were sacrificed with an overdose of sodium pentobarbital. Eyes were quickly removed after a reference point was made to label the superior pole. For analysis of outer nuclear layer (ONL), eyeballs were removed and fixed in 4% paraformaldehyde (PFA) in 0.1 M phosphate buffer, pH 7.4 overnight at 4°C. Samples were dehydrated with a graded series of ethanol and xylene and subsequently embedded in paraffin wax as previously described by us and other group^11^,^17^. Retinal sections (5 μm) were cut through the corneal center and the center of the optic nerve and stained with hematoxylin and eosin (h&e). Thickness of the ONL, which contains the photoreceptor nuclei, was measured to assess rod photoreceptor survival. We performed measurements on these retinal sections that passed through the optic nerve head. Because the ONL thickness and the degenerative process in the rd10 model are not uniform and dependent upon location, all measurements were performed in the mid-peripheral retinal region at 1 mm from the optic nerve head. For some animals, retinal flat-mounts were prepared after removing the eyeball. Retinas were dissected in carboxygenated Ames' Medium (Sigma-Aldrich, St. Louis, MO), and then fixed in 4% PFA for 1 hour. Both whole-mounted retinas and vertical sections were blocked in a solution containing 3% normal donkey serum (NDS), 1% bovine serum albumin (BSA), and 0.3% Triton X-100 in PBS (pH 7.4) for 1 hour. Primary antibodies used were rabbit antibody to GFP (1:500, Invitrogen, Carlsbad, CA), rabbit anti-red/green opsin (1:500, Chemicon, Temecula, CA), and rat anti-mouse CD68 (1:500, AbD Serotec, Raleigh, NC). Retinas were incubated in a primary antibody, which was diluted with a blocking solution (1% NDS, 1% BSA, 0.1% Triton X-100 in PBS), for overnight to 3 days at 4°C. After blocking and rinsing, a secondary antibody conjugated to either Alexa 488 (1:500; Invitrogen) or Alexa 594 (1:500; Invitrogen) was applied for 2 hours at room temperature. Retinas were rinsed, and cover slipped in Vectashield mounting medium (Vector Laboratories, Burlingame, CA).

Confocal micrographs of fluorescent specimens from retinal flat-mounted preparations and vertical sections were captured using a Zeiss LSM 700 Meta Axioplan 2 laser scanning confocal microscope (Carl Zeiss, Oberkochen, Germany) equipped with argon and helium-neon lasers. Plan-Apochromat 40×/1.4 or 63×/1.4 oil immersion objectives were used.

### Electroretinographic (ERG) analysis

ERGs were recorded with an Espion ERG Diagnosys machine (Diagnosys, Littleton, MA) as previously described by us^18^. In brief, mice were anaesthetized intraperitoneally with a mixture of Dormitor (1 mg/kg medetomidine hydrochloride; Pfizer, UK) and Ketamine after overnight dark adaption. Pupils were dilated with 1% Mydriacyl (Alcon, Ontario, Canada). Flash ERG was measured using a gold wire corneal electrode, a forehead reference electrode, and a ground electrode near the tail.

Scotopic, rod-mediated responses were obtained from dark-adapted animals at the following increasing light intensities: 0.01 and 3 cd-s/m^2^. Photopic, cone-mediated responses were performed following 10-minute light adaptation on the background light intensity of 30 cd/m^2^. Recordings were obtained at the light intensity of 3 cd-s/m^2^. The ERG a-wave amplitudes were measured from the baseline to the negative peak and the b-wave was measured from the trough of the a-wave to the peak of the positive wave or, when the a-wave was not present, from baseline to the peak of the first positive wave.

### Optokinetic tracking

Optokinetic tracking was performed as previously described by us^18^. This test measures the tendency of an animal to follow with the head the movements of the surrounding environment. In practice, it was tested by placing the animal on a platform positioned in the middle of an arena created by a quad-square of computer monitors after overnight dark adaptation. Vertical sine wave gratings (100% contrast) were projected on computer monitors. The spatial frequencies tested were 0.05, 0.1, 0.2, 0.3, 0.4, 0.5, and 0.6 cycle per degree (cpd), at a constant speed of 12 degree per second. Images of head movements were monitored using an infrared-sensitive small camera.

### Enzyme-linked immunosorbent assay (ELISA) measurement

ELISA for TNF-α, Il-6β, CCL2, HIF-1α, caspase-3/7, and Bax levels in retinas were quantified using ELISA kits from PeproTech (Rocky Hill, NJ), Cell Signaling (Danvers, MA), and EIAab (Wuhan, China), respectively, according to manufacturer's instructions as previously descripted by us^18^,^19^. The activity of transcription factor NF-kB p65 was measured with a NF-kB/p65 ActivELISA kit (IMGENEX Corporation, San Diego, CA) from nuclear extracts according to user's instructions. The ratio of the optical density of the protein product to the internal control (β-actin) was obtained and was expressed as ratio of the control value in the Figures using ImageJ software (NIH, Bethesda, MA).

### Western blot analysis

CD68, phospho-IκBα, and IκBα proteins were detected by Western blotting as previously described by us^18^,^19^. Protocols for cytosolic and nuclear protein extraction were performed as previously described^20^. The ratio of the optical density of the protein product to the internal control (β-actin) was obtained and was expressed as a ratio or percentage of the control value in the Figures.

### Data analysis

For measurement of the ONL thickness, vertical sections passing through the optic nerve head were analyzed by counting the number of photoreceptor nuclei stained with h&e. Two measurements were taken at 1 mm from the optic nerve on both sides along the dorsal-ventral axis. Quantification of surviving cones stained with red/green opsins was conducted in retinal whole-mounts. Sampling areas were two 240 μm × 240 μm squares along the dorsal-ventral axis of retinal whole-mounts per retina, 1 mm from the optic nerve on both sides. The raw counts were then converted into cells/millimeter^2^.

### Statistical analyses

All data were expressed as either means ± SEMs or means ± SDs. ANOVAs with Bonferroni's and Dunnett's *post hoc* tests for multiple comparisons were performed with Origin (OriginLab) and programs written in MATLAB (Mathworks) on full data sets to detect significant differences in the means. A p value < 0.05 was considered statistically significant.

## Results

### Polysaccharides preserved photoreceptor morphology in the rd10 retina

**Figure 1:**
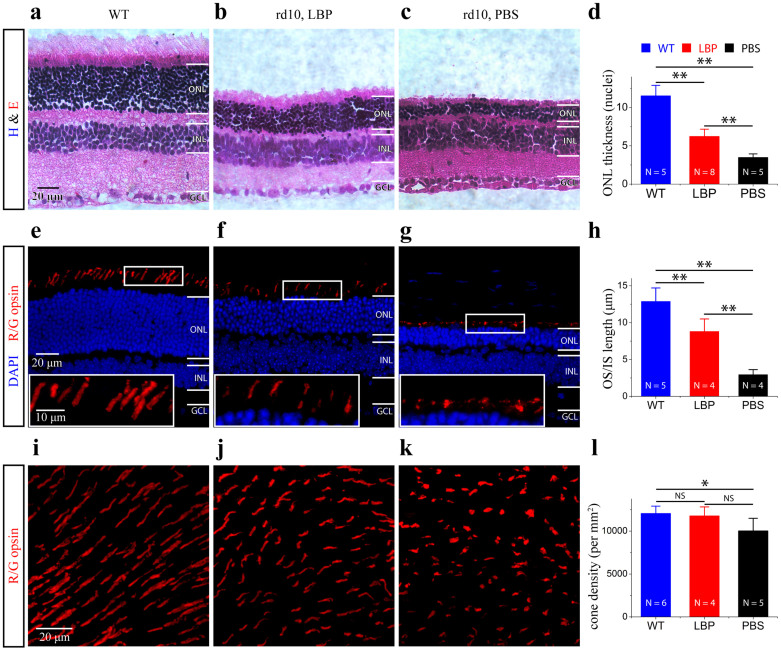
Polysaccharides preserved photoreceptor morphology in the rd10 retina at P26. (a–c). Representative photomicrographs of hematoxylin and eosin (h&e) stained ultra thin retinal sections. The thinner ONL is observed in PBS-treated rd10 retina (c), while polysaccharides preserved the thickness of the ONL (b). Retinas from WT mice at the same age are used as a comparison (a). (d). Quantification of the ONL thickness, measured in numbers of photoreceptor nuclei per column. Results are presented as means ± SDs; n: number of retinal sections in each group. ** p < 0.01. (e–g). The OS/ISs of cones are revealed by an antibody against red/green opsin (red). Cone's OS/ISs in polysaccharides- treated rd10 mice ((f), red) appear longer than those in PBS-treated rd10 mice ((g), red). Insets illustrate highly magnified image of the OS/ISs of cones from the boxed regions. (h). Plot of the length of OS/ISs. Results are presented as means ± SDs; n: number of retinal sections in each group. ** p < 0.01. (i–k). Retinal whole-mounts. Confocal images of cone from the superior region of the retina of WT (i), polysaccharides- (j), and PBS- treated rd10 mice (k) are shown. (l). Quantification of cone density. Results are presented as means ± SDs; n: number of flat mounted retinas in each group. ns, not significant, * p < 0.05. ONL: outer nuclear layer; INL: inner nuclear layer; GCL: ganglion cell layer; OS: outer segment; IS: inner segment.

We investigated the neuroprotective effect of polysaccharides on photoreceptor death in the rd10 mouse model of RP, which is caused by a mutation in the rod-specific gene that encodes rod cGMP phosphodiesterase β-subunit (Pde6β)^21^. Rod photoreceptors start degeneration around postnatal day 18 (P18) in rd10 retinas^15^. We thus fed rd10 mice with polysaccharides from P14, a few days earlier before the initiation of rod degeneration. Morphological analysis was performed after 12-day treatments at P26. Because roughly 97% of photoreceptor nuclei in the outer nuclear layer (ONL) of the mouse retina are rods^22^, we thus used the ONL thickness as an index for the assessment of rod death. We counted the rows of photoreceptor nuclei in the ONL on retinal vertical sections (Figure 1 a–c). At P26, the ONL became dramatically thinner (by 69.6%) in rd10 retinas (3.4 ± 0.5 nuclei/column, Figure 1c), compared with normal C57/BL6J retinas (11.5 ± 1.3 nuclei/column, p < 0.01; Figure 1a, d). Polysaccharides-treated rd10 retinas had significantly thicker ONL (by 77.1%) (6.2 ± 1.0 nuclei/column, Figure 1b) than in PBS-treated eyes (p < 0.01; Figure 1d), indicating that polysaccharides reduced rod cell death.

Moreover, polysaccharides treatment slowed down the progression of cone degeneration in rd10 mice. In WT retinas, the outer segments of cones were stained with antibodies specific for red/green cone opsins (Figure 1e, red). However, the morphology of cone outer segments/inner segments (OS/IS) was disrupted in rd10 controls (Figure 1g, red). The OS/ISs of cones were largely maintained in polysaccharides-treated rd10 retinas (Figure 1f, red). Qualification of the OS/IS length on retinal sections showed that up to 54.1% of the normal outer segment length was maintained in the polysaccharides-treated rd10 retina whereas less than 23.2% of the normal outer segment length remained in rd10 controls when compared to WT retinas (12.4 ± 1.4 μm in C57/BL6J retinas; 6.7 ± 1.7 μm in polysaccharides -treated rd10 retinas; 2.5 ± 0.3 μm in PBS-treated rd10 retinas; p < 0.01; Figure 1h). The similar trend was observed when we quantified the OS/IS length from flat mounted retinas (12.9 ± 1.8 μm in C57/BL6J retinas; 8.8 ± 1.7 μm in polysaccharides-treated rd10 retinas; 3.0 ± 0.7 μm in PBS-treated rd10 retinas; p < 0.01).

**Figure 2:**
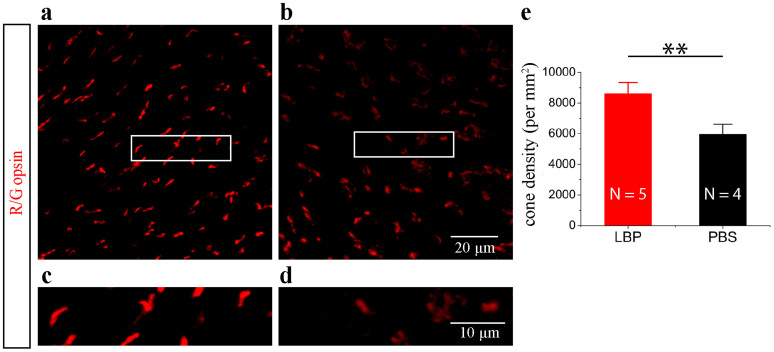
Polysaccharides promoted cone photoreceptor survival in the rd10 retina at P30. (a–b). Confocal images of retinal wholemounts. Cone photoreceptors are revealed by an antibody against red/green opsin (red). Polysaccharides-treated rd10 mice (a) appeared to have greater cone density compared to PBS-treated mice (b). (c–d). Illustrate highly magnified image of the OS/ISs of cones from the boxed regions above, respectively. (e) Quantification of cone density. Results are presented as means ± SDs; N: number of flat mounted retinas in each group. ** p < 0.01.

We quantified cone density on flat mounted retinas from three groups of mice at P26 (Figure 1 i–k). We found that cone density in PBS-treated rd10 mice was significantly lower compared with WT, while there was no difference between WT and polysaccharides-treated rd10 mice in cone density (WT retinas: 12043.9 ± 870.8 cones/mm^2^; polysaccharides-treated rd10: 11757.5 ± 1058.6 cones/mm^2^; PBS-treated rd10: 10014.7 ± 1476.0 cones/mm^2^, Figure 1l). A higher cone density was also observed in polysaccharides-treated rd10 mice compared with PBS-treated rd10 mice, but did not attain statistical significance (p > 0.05; Fig. 1l). Soon after that, however, we observed that treatment by polysaccharides delayed the progression rate of cone degeneration when compared with PBS-treated rd10 mice (Figure 2 a, b). The density of cone photoreceptors was significantly higher in polysaccharides-treated rd10 mice at P30 (by 44.7%) than PBS-treated rd10 mice (8627.0 ± 709.0 cones/mm^2^ in polysaccharides-treated rd10 retinas; 5961.3 ± 655.0 cones/mm^2^ in PBS-treated rd10 retinas; p < 0.01; Figure 2e). Therefore, polysaccharides provided a significant morphological preservation of rods and cones in rd10 retinas.

### Polysaccharides preserved photoreceptor function in the rd10 retina

**Figure 3:**
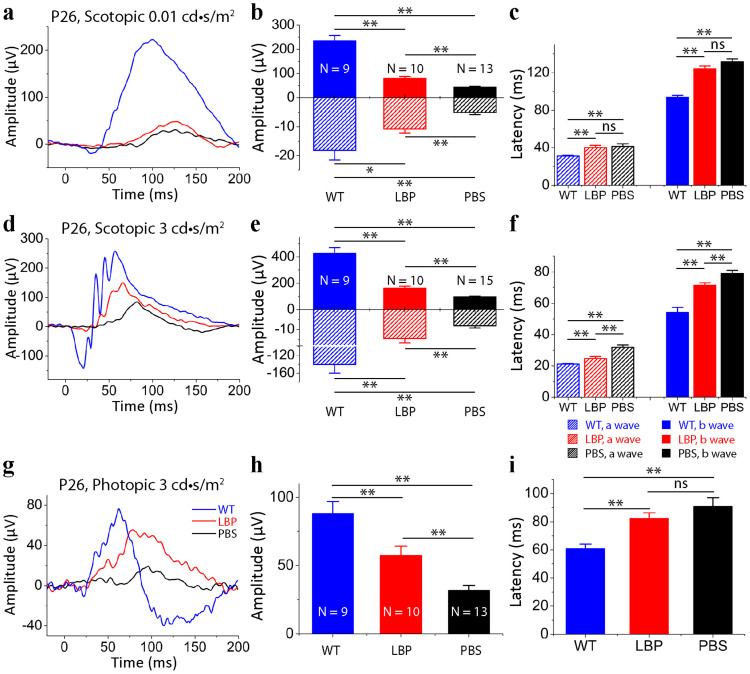
Polysaccharides preserved photoreceptor function in the rd10 retina. (a), (d). Representative scotopic ERG responses to 0.01 (a) and 3 cd-s/m^2^ (d) light intensities from P26 rd10 mice treated with polysaccharides (red curve) or PBS (dark curve) from P14 to P25. Scotopic ERG responses from age-matched C57BL/6J (blue curve) are shown as comparisons. (b), (e). Average scotopic a- and b-wave amplitudes elicited at 0.01 cd-s/m^2^ (b) and 3 cd-s/m^2^ (e) light intensities. Since a waves of ERGs were negative, we presented a-wave amplitudes on the lower, negative axis. (g). Representative photopic ERG responses to 3 cd-s/m^2^ light intensity from rd10 mice at P26. (h). Averaged photopic b-wave amplitudes elicited at 3 cd-s/m^2^ light intensity from normal C57 BL/6J (blue), polysaccharides- (red curve) or PBS-treated (dark) rd10 mice. (c), (f), (i). Average latency time (time to peak) for scotopic ERG a- and b-waves at 0.01 cd-s/m^2^ (c) and 3 cd-s/m^2^ (f) light intensities, and for photopic ERG b-waves at 3 cd-s/m^2^ (i). Data are expressed as the means ± SEMs. n: number of animals in each group. ns, not significant, *: p < 0.05, ** p < 0.01.

To examine functional preservation in rd10 mice, we performed electroretinography (ERG) on mice at P26. Figure 3 shows representative ERG recordings from polysaccharides- and PBS-treated rd10 mice (Figure 3 a, d, g). The scotopic a- and b-wave amplitudes in polysaccharides-treated rd10 mice were small but significantly higher than those in PBS-treated rd10 mice (p < 0.01; Figure 3 a–b, d–e). Photopic ERG b-wave amplitudes were similarly larger compared to PBS-treated rd10 controls (p < 0.01; Figure 3 g–h).

In addition, the latency of a- and b-waves under scotopic and photopic conditions was measured in polysaccharides-treated rd10 mice. Latency time for b-waves differed between polysaccharides- and PBS-treated rd10 mice under both dark and light-adapted conditions (p < 0.01; Figure 3 c, f, i). We found no significant difference in latency time for a-waves at 0.01 cd-s/m^2^ under dark-adapted condition and at 3 cd-s/m^2^ under light-adapted condition (p > 0.05, Figure 3 c, i). However, a significant difference in latency time was observed at 3 cd-s/m^2^ under dark-adapted condition (p < 0.01, Figure 3f).

### Polysaccharides improved visual performance in rd10 mice

**Figure 4:**
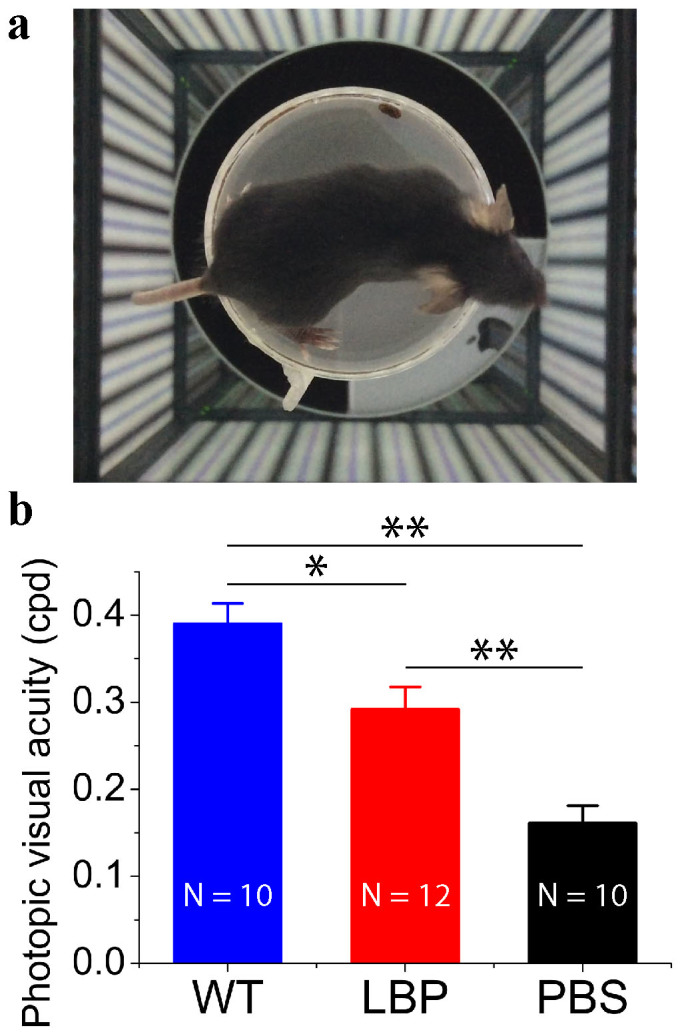
Polysaccharides improved visual acuity in rd10 mice. Photopic visual acuity was measured by the optokinetic response after overnight dark adaptation. (a). A single-frame video camera image of a mouse surrounded by 360° of sine wave gratings. (b). PBS-treated rd10 mice show much poorer acuity (black bar) compared to WT eyes (p < 0.01; blue bar). Visual acuity in polysaccharides-treated rd10 mice (red bar) is better than PBS-treated rd10 mice (P < 0.01; orange bar), but is still poorer than WT mice (p < 0.01; blue bar). Results are presented as means ± SEMs; n: number of animals in each group. *: p < 0.05, **: p < 0.01.

To examine the spatial visual performance in rd10 mice after polysaccharides treatment, we measured optomotor responses to moving gratings at 100% contrast (Figure 4a). At P26, photopic visual acuity decreased dramatically in rd10 mice compared to WT mice (P < 0.01; Figure 4b). Polysaccharides-treated rd10 mice exhibited improved acuity. Light-adapted visual acuity was approximately 2-fold higher in polysaccharides-treated rd10 mice than that in PBS-treated rd10 mice (P < 0.01; Figure 4b), although a statistical difference in visual acuity was also found between WT mice and polysaccharides-treated rd10 mice (P < 0.05; Figure 4b). Thus, polysaccharides treatments improved visual behavior in rd10 mice.

### Polysaccharides suppressed microglia activation in the rd10 retina

**Figure 5:**
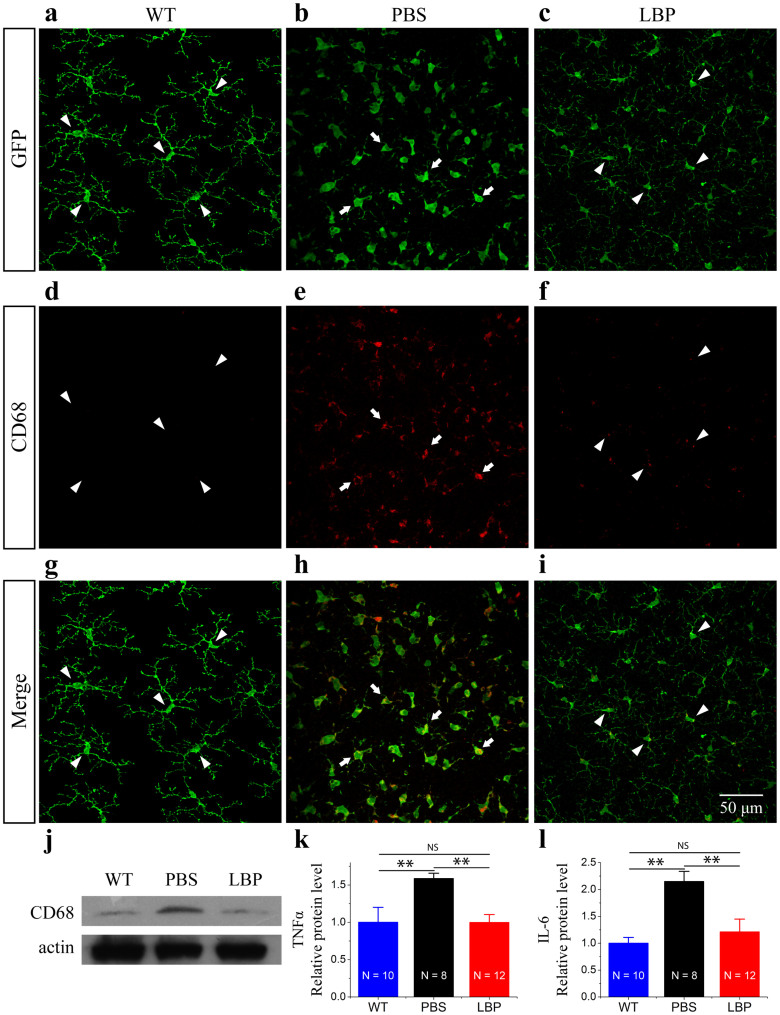
Polysaccharides reduced microglia activation in rd10/*Cx3cr1^+/GFP^* mouse retinas. (a–c). Microglia have a ramified morphology in WT retinas ((a), arrowheads), while microglia show an ameboid shape in rd10 retinas at P26 ((b), arrows). After polysaccharides treatment, microglia maintained a ramified morphology in rd10 retinas ((c), arrowheads). (d–f). CD68 staining is undetectable in WT retinas ((d), arrowheads), and CD68 staining is intense and widespread in rd10 retinas ((e), arrows). However, CD68 staining is reduced in polysaccharides-treated rd10 retinas ((f), arrowheads). (g–i). Merged images. (j). Western blot of CD68 protein expression in the retina of WT, polysaccharides- and PBS-treated rd10 mice. (k–l). Analysis of TNF-α (k) and IL-6β (l) expressions in the retina of WT and polysaccharides- and PBS-treated rd10 mice by ELISA. N, number of retinas in each group. Results are presented as means ± SDs, ns, not significant, **: p < 0.01.

In WT *Cx3cr1^+/GFP^* mouse retinas, microglial cells had a ramified shape (Figure 5a, arrowheads). However, these cells underwent a gradual morphological change from a ramified appearance into an amoeboid shape in rd10/*Cx3cr1^+/GFP^* mouse retinas (Figure 5b, arrows), indicative of an activated state. Ramified microglial cells were maintained after polysaccharides treatment (Figure 5c, arrows) indicative of an inactivated state. In addition, we performed CD68 staining, a marker for microglia activation, to confirm the state of microglial activation. Microglial cells were negative for CD68 immunoreactivity in WT retinas (Figure 5 d, g), whereas CD68 immunoreactivity was strong and intense in the rd10 retina (Figure 5 e, h, arrows). After polysaccharides treatment, CD68 immunoreactivity was reduced in the rd10 retina (Figure 5 f, i). Western blot analysis confirmed the results from immunohistochemistry (Figure 5j). CD68 expression level increased 6.8-fold higher in rd10 retina compared to WT retinas, whereas CD68 expression level was 6.2-fold lower in polysaccharides-treated rd10 retinas compared to PBS-treated rd10 controls (Figure 5j).

**Figure 6:**
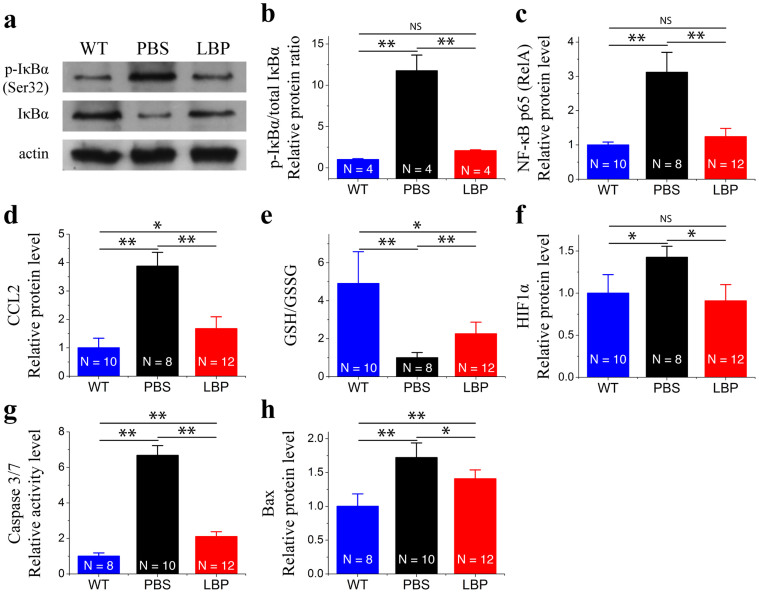
Polysaccharides downregulated expression levels of apoptosis-related molecules. (a–b). Western blot of IκBα, phospho-IκBα expressions in the retina of polysaccharides- and PBS-treated rd10 mice and WT mice. (c). NF-κB p65 expression in the retina of WT and polysaccharides- and PBS-treated rd10 mice by ELISA. (d). Analysis of CCL2 expression in the retina of WT and polysaccharides- and PBS-treated rd10 mice by ELISA. (e). GSH and GSSG expression in the retina of WT and polysaccharides- and PBS-treated rd10 mice by ELISA. (f–h). HIF1α (f), Caspase-3/7 (g), and BAX (h) are similarly upregulated in rd10 retinas (black bars), and downregulated following polysaccharides treatment (red bars). Results are presented as means ± SDs. N, number of retinas in each group. ns, not significant, * p < 0.05, **: p < 0.01.

To find out whether suppression of microglial activation led to the reduction in production of inflammatory mediators, we evaluated the expression levels of TNF-α, Il-6β and CCL2. TNF-α and Il-6β expressions significantly increased in rd10 retinas compared to WT retinas (p < 0.01; Figure 5 k, l). The expression levels of these inflammatory mediators were reduced with polysaccharides treatment (p < 0.01; Figure 5 k, l). Similar trend was observed for CCL2 (p < 0.01; Figure 6d), indicating attenuation of neuroinflammation in polysaccharides-treated rd10 retinas.

Seeking to determine the underlying mechanism of inhibition of microglia activation, we studied the expression level of nuclear factor-κB (NF-κB) in the nucleus and inhibitor of nuclear factor-κB (IκB) in the cytoplasm. Phosphorylated-IκBα increased 4.5-fold in PBS-treated rd10 retinas compared to WT retinas (Figure 6 a, b), and polysaccharides treatments significantly reduced phosphorylated-IκBα in rd10 retina by 3.8-fold (Figure 6 a, b). On the other hand, IκBα expression decreased 6-fold in PBS-treated rd10 retinas compared to WT retinas, and the expression level of IκBα increased 5-fold in polysaccharide-treated rd10 retinas compared to PBS-treated rd10 retinas (Figure 6 a, b). The ratio of phosphorylated- IκBα/total- IκBα in PBS-treated rd10 retinas was 8.9-fold higher than that in WT retinas (p < 0.01, Figure 6 a, b). On the other hand, there was no difference in the ratio between polysaccharides-treated rd10 mice and WT mice (p > 0.05, Figure 6 a, b). NF-κB induces the transcription of proinflammatory genes in the nucleus^23^,^24^,^25^. We observed that phosphorylated-NF-kB p65 (p-NF-kB p65) increased approximately 3-fold in rd10 retinas compared to WT retinas (p < 0.01; Figure 6c). Polysaccharides treatment significantly reduced p-NF-kB p65 expression level in rd10 retinas (p < 0.01; Figure 6c). Our data suggested that polysaccharides mediated the expression levels of inflammatory mediators partially through the NF-κB signaling pathway.

### Polysaccharides targeted several underlying mechanisms in the rd10 retina

To understand the underlying mechanisms, we further investigated whether polysaccharides exerted its protective effect via antioxidation and anti-apoptosis. Glutathione redox/antioxidant (GSH/GSSG) ratio is commonly used in measuring oxidative stress status, and a reduction in GSH/GSSG ratio reflects a reduced antioxidant capacity. Compared to WT retinas, the GSH/GSSG ratio in rd10 retinas was significantly reduced (p < 0.01; Figure 6e). Polysaccharides increased the GSH/GSSG status in rd10 retinas compared with rd10 controls (p < 0.01; Figure 6e), indicating increased antioxidant capacity.

Moreover, the upregulation of hypoxia-inducible factor 1α (HIF-1α) is observed in rd mouse models of RP^26^. Similarly, we found the upregulation of HIF-1α in rd10 retinas compared to WT retinas (p < 0.05; Figure 6f), and the increase was counteracted by polysaccharides (p < 0.05; Figure 6f). Caspase-3/7 activity level increased more than 6-fold in rd10 retinas compared to WT controls at P26 (p < 0.01; Figure 6g). Similarly, Bax expression significantly increased in rd10 retinas (p < 0.01; Figure 6h). Following 12-day polysaccharides treatment, we observed a significant reduction in expression levels of activated caspase-3/7 and Bax in rd10 retinas (Figure 6 g, h).

Together, our data suggested that the protective effects of polysaccharides were mediated partially through inhibition of oxidative stress and apoptosis, leading to photoreceptor survival in rd10 mice.

### Polysaccharides provided long-term protection of photoreceptors in the rd10 retina

**Figure 7:**
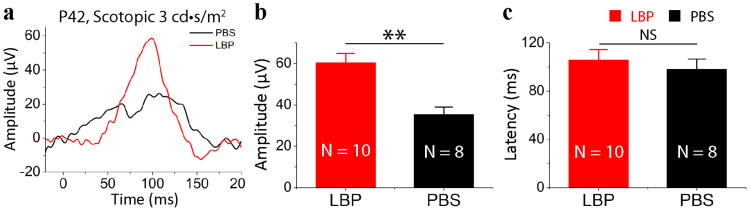
Polysaccharides exerted long-term functional protection of photoreceptors in the rd10 retina. (a). Representative scotopic ERG responses to 3 cd-s/m^2^ light intensity from P42 rd10 mice treated with polysaccharides (red curve) or PBS (dark curve) from P14 to P41. (b). Average scotopic b-wave amplitudes. (c). Average latency time (time to peak) for scotopic ERG b-waves. Values are means and SEMs. N, animal numbers in each group; ns, not significant, and ** p < 0.01.

To investigate long-term protective effect of polysaccharides on photoreceptor apoptosis, we treated rd10 mice for 28 days from P14 to P41. Remarkably, we found that the protective effect of polysaccharides was still profound after treatment at P42 (Figure 7). Although dark-adapted a-waves became undetectable to 3 cd-s/m^2^ light intensity in all rd10 animals, dark-adapted b-wave amplitudes in polysaccharides-treated rd10 mice were approximately twice as large as that in PBS-treated groups (p < 0.01; Figure 7 a, b). On the other hand, latency time for b-waves did not significantly differ between polysaccharides- and PBS-treated rd10 mice (p > 0.05; Figure 7c). Thus, polysaccharides provided long-term functional preservation of photoreceptors in rd10 retinas.

## Discussion

Retinitis pigmentosa (RP) is a group of hereditary retinal diseases, in which progressive degeneration of photoreceptors occurs. It is well known that mutations of rod-specific genes lead to rod photoreceptor death, ultimately resulting in the secondary cone degeneration and complete blindness in RP. Even though the underlying genetic mutations of RP are known, the exact mechanisms by which the mutations lead to photoreceptor death are still not completely understood. The disease with enormous genetic heterogeneity, such as RP, poses a problem in the development of treatments that deals with primary genetic defects. Attacking common downstream pathways leading to photoreceptor degeneration may provide meaningful benefit to most RP patients. To this end, we explored the potential neuroprotective effects of *Lycium barbarum* polysaccharides (LBP), whose primary protective function is in the eyes, on photoreceptor apoptosis in the rd10 mouse model of RP. We showed that polysaccharides reduced photoreceptor cell death rate, improved scotopic and photopic ERG responses, and enhanced spatial visual performance in rd10 mice. Remarkably, polysaccharides were effective in maintaining photoreceptor morphology and function through the advanced degenerative time point of P42 rd10 mice. Moreover, we identified that the neuroprotective effect of polysaccharides was correlated to inhibition of microglial activation, oxidative stress, and photoreceptor apoptosis.

Microglia activation is associated with photoreceptor degeneration in human RP and mouse models of RP^27^,^28^,^29^. Previous studies have shown that microglial activation occurs in the early phase of retinal degeneration in animal models of RP^28^,^29^. Subsequently, increased expression of proinflammatory cytokines and chemokines precedes the occurrence of photoreceptor apoptosis in animal models of RP^29^,^30^. Moreover, we have recently shown that suppression of microglia activation promotes photoreceptor survival in the rd10 mouse model of RP^18^, confirming the pathological role of microglia in photoreceptor apoptosis. Consistent with prior findings, we observed microglial activation by immunohistochemistry and Western blot analysis, and increased expression of proinflammatory mediators by ELISA analysis in the rd10 mouse retina. After polysaccharides treatment, we observed the correlation between the downregulation of the expression levels of CD68, TNF-α, Il-6β and CCL2 and amelioration of photoreceptor apoptosis in rd10 retinas. We further demonstrated that NF-kB inhibition was likely to be the mechanism by which proinflammatory mediators were down-regulated. Consistently, evidences from our previous studies in the liver indicated that polysaccharides suppress acute and chronic hepatic cellular inflammation partially through modulating the NF-κB^31^,^32^,^33^. Our previous studies in the liver demonstrated the main underlying key signaling events such as inflammation, oxidative stress and apoptosis. At least two of the central sensors and regulators of these events are involved such as NF-κB and HIF-1α. We thereby extended our investigation to evaluate the expressions of relevant down-stream molecules related to these events. This rationale was also applied in this retinal study because of similarity of main pathways occurring in both systems.

Accumulating evidence has shown that oxidative stress is another pathogenic factor that contributes to photoreceptor cell death in RP. Administrations of antioxidants^3^,^34^ and iron chelators^35^,^36^ reduce oxidative stress, and attenuate photoreceptor apoptosis in mouse models of RP. Similarly, we found that polysaccharides counteracted oxidative challenges by restoring antioxidant enzymes, leading to attenuation of collateral oxidative stress injury and enhancement of photoreceptor survival. However, we did not exclude other factors/mechanisms that might also be involved in the inhibition of oxidation. Our previous studies have reported that the neuroprotective effects of polysaccharides on the survival of retinal ganglion cells are related to the dowreagulation of superoxide dismutase-1 (SOD-1) in retina after optic nerve transection^16^.

Hypoxia-inducible factor 1α (HIF-1α) regulates many biological processes and has been shown to activate the transcriptional activity of p53, which leads to transcription of many pro-apoptotic proteins, such as BAX and caspase-3^37^,^38^. The upregulation of HIF-1α and caspase-3 is observed during photoreceptor degeneration in rd mouse models of RP^26^,^39^. Downregulation of HIF-1α or caspase-3 delays photoreceptor death RP mice^26^,^40^. Similarly, we found increased expressions of HIF-1α, activated caspase-3/7 and Bax in rd10 retinas, which was downregulated by polysaccharides. Polysaccharides probably suppressed the expressions of activated caspase-3/7 and Bax partly through the inhibition of HIF-1α expression in rd10 retinas. Our earlier studies show that polysaccharides protect against the receptor for advanced glycation end products (RAGE), amyloid-β (Aβ) peptide neurotoxicity and advanced glycation end products (AGEs) in ischemic retinas, and inhibit pro-apoptotic signaling pathways such as the c-jun N-terminal kinase (JNK) pathway in the retina after the optic nerve transection^11^,^16^. On the other hand, polysaccharides can up-regulate the expression of insulin-like growth factor-1 (IGF-1) on retinal ganglion cells, which are helpful to the survival of RGCs in the retina after partial optic nerve transection^16^. The present study extended overall the understanding of anti-apoptotic mechanisms by identifying more signaling pathways targeted by polysaccharides.

In summary, we demonstrated the synergistic protective effects of polysaccharides in preserving photoreceptors against degeneration in the rd10 mouse model of RP. Polysaccharides treatment preserved photoreceptor structure, function and visual behavior in rd10 mice, even though polysaccharides could not fully arrest degenerative processes caused by genetic mutations. Similarly, our previous studies show that polysaccharides exerted neuroprotective effects through multiple pathways including anti-oxidation, anti-inflammation, and anti-apoptosis in other ocular^11^,^16^ and liver disease models^31^,^32^,^33^. Our present data provided additional support for the hypothesis that attacking common downstream pathways leading to neurodegeneration, rather than targeting numerous primary genetic defects, is a sensible strategy for the treatment of neurodegenerative diseases. Considering its low toxicity and wide availability, our study provides scientific support and rationale on using polysaccharides of wolfberry as one supplementary treatment for RP patients in the future.

## Author Contributions

K.W., B.P. and B.L. designed the study. K.W., J.X. and B.P. performed the experiments. K.W., J.X., B.P., F.X., K.F.S., G.L.T. and B.L. analyzed the data. G.L.T. and B.L. wrote and edited the manuscript. All authors reviewed and approved the manuscript.
